# Advances in the Diagnosis of Atypical Polypoid Adenomyoma Combining Immunohistochemical and Molecular-Based Approaches: Case Report and Review of the Literature

**DOI:** 10.3390/cimb46120850

**Published:** 2024-12-17

**Authors:** Francisco Javier Azúa-Romeo, Sonia Bayona-Morón, Irene Rodriguez-Perez, Bárbara Angulo-Biedma

**Affiliations:** 1 Department of Pathology, Analiza, 28001 Madrid, Spain; irodriguez@analizalab.com (I.R.-P.); bangulo@analizalab.com (B.A.-B.); 2 Department of Anatomy and Histology, Faculty of Medicine, University of Zaragoza, 50009 Zaragoza, Spain; senayo-3@hotmail.com

**Keywords:** atypical polypoid adenomyoma, endometrial cancer, immunohistochemistry, molecular biology

## Abstract

Atypical polypoid adenomyoma (APA) is a benign uterine lesion with a premalignant potential and occurs in women of reproductive age. The histological pattern is characterized by irregular epithelial proliferation and muscular stroma. Based on a case report, we performed a systematic review of the literature to assess the main immunohistochemical and molecular markers that contribute to its differential diagnosis against endometrial adenocarcinoma (EC). The distinction is essential for offering to patients a conservative treatment compared to the radical management required for endometrial cancer, a critical issue for the significant physical and psychological consequences that one procedure or another can have on women’s health. We performed a meta-analysis of the immunohistochemical markers used for the histological diagnosis of APA, comparing it with our case study. The evaluated markers were beta-catenin, h-caldesmon, desmin, vimentin, smooth muscle alpha-actin, CD10, Ki67, estrogen receptor (ER), progesterone receptor (PR), pan-cytokeratin, PTEN, PMS2, MSH2, MSH6, p53, MLH1, and p16. Discrepancies were observed in the expression of CD10, h- caldesmon, and p16 when comparing APA with EC. The results of the case evaluated by our team showed beta-catenin nuclear expression and positive immunostaining for pan-cytokeratin, ER, and PR in the glands; smooth muscle actin and desmin positive expression in stromal muscle; and p16 positive immunostaining in squamous morules. Moreover, the c.94G>T p. (Asp132Tyr) mutation in the *CTNNB1* gene was detected. This study supports the combination of appropriate immunohistochemical and molecular markers, along with the presumptive histological diagnosis, and determines the correct classification of the lesion as APA and not as other malignant pathologies, allowing for the establishment of a treatment protocol adjusted to the biological reality of this pathology.

## 1. Introduction

Atypical polypoid adenomyoma (APA) is a rare lesion primarily located in the uterine body, mostly occurring in premenopausal women with a mean age of 39.30 ± 11.01 years, ranging from 17 to 64 years, and an average body mass index of 27.63. It manifests with abnormal vaginal bleeding, dysmenorrhea, polyps, and even infertility or asymptomatic clinical courses, being an incidental finding in infertility studies or abnormal cytology [1,2]. Histologically, it is characterized by irregular proliferation of endometrial glands, which can be tubular or have complex branching, sometimes containing squamous morules with a central focus of necrosis. This epithelial tissue can be arranged in clustered or more spaced configurations, surrounded by smooth fibromuscular stroma organized in short, interlaced bundles with minimal cellular atypia and sporadic mitotic cells [3]. Other studies have detected mild cytological atypia in most APA (97%) and endometrioid endometrial carcinomas (EECs), 97% and 93%, respectively, with a higher mitotic rate in the latter [4].

APA was included in the classification of mixed epithelial and mesenchymal tumors of the uterus, along with choriocarcinoma, adenosarcoma, adenofibroma, and adenomyoma, by the World Health Organization (WHO) in 2014 [5]. First described by Mazur in 1981 and considered benign at that time, subsequent studies have observed some potential for malignancy; however, more extensive research is required to substantiate this hypothesis [6]. According to other authors, there is a medium risk of endometrial carcinoma (EC) in women with atypical polypoid adenomyoma, estimated at 8.8% [7]. The literature reveals that APA lesions share immunohistochemical and molecular characteristics with EC, such as loss of PTEN expression and *KRAS* mutations. It has been proposed that APA could be considered analogous to a localized form of atypical endometrial hyperplasia [8].

For diagnosing this non-specific presentation of abnormal uterine bleeding, the initial approach is based on anamnesis and physical and gynecological examination. The first-line imaging test for structural lesions is transvaginal ultrasound, complemented by hysteroscopy with endometrial biopsy for structural causes. Due to the architectural complexity of the lesion, histopathological study using immunohistochemistry (IHC) and molecular pathology techniques is necessary for a proper differential diagnosis with the most common histological type, EEC, which is mostly low-grade and glandular. EC is the most common malignant gynecological tumor in Spain and the second in mortality after ovarian cancer. Therefore, it is essential to individualize treatment based on age, clinical presentation, and malignancy potential. In women of childbearing age with reproductive desire, conservative treatment through hysteroscopic resection or even medical treatment with progestogens is advisable due to the estrogen-dependent nature of the pathology, compared to total hysterectomy with bilateral adnexectomy performed as the surgical treatment of choice for endometrial cancer. Chemotherapy, external radiotherapy, and brachytherapy are reserved for inoperable patients or as adjuvant therapy if there is intermediate, high, or advanced risk [9,10]. For example, Rizzuto et al. [11] described the case of a pregnant woman incidentally diagnosed with endometrial adenocarcinoma within an APA and treated conservatively, leading to a successful term pregnancy.

Advanced immunohistochemistry and molecular pathology techniques were used for lesion diagnosis, crucial for the precise detection of specific biomarkers. Samples were processed using standardized protocols for fixation, paraffin embedding, and sectioning. Immunohistochemical techniques were applied using specific primary antibodies for markers such as b-catenin, h-caldesmon, desmin, vimentin, smooth muscle alpha-actin (SMA), CD10, ki67, ER, PR, pancytokeratin, PTEN, PMS2, MSH2, MSH6, P53, MLH1, and P16, chosen for their relevance in identifying pathological features of the analyzed lesion. The interpretation of these findings highlights the importance of using specific markers in the precise diagnosis of lesions, providing a solid basis for appropriate therapeutic decisions.

In the work reported here, we presented a detailed description of a case involving a 40-year-old female patient with abnormal uterine bleeding. Following a myomectomy and total polypectomy, a histopathological diagnosis revealed the presence of an APA. Based on our diagnostic process for this patient, and performing an exhaustive review of the literature, our goal is to provide the tools for an adequate diagnosis of APA, combining clinical and histopathological findings and complementary imaging tests. All of these factors could contribute to guide clinical management, establish patient prognosis, and minimize the emotional impact associated with the disease.

## 2. Case Description

### 2.1. Literature Search and Selection

For this study, a comprehensive bibliographic review was conducted using PubMed and Google Scholar databases, selecting articles published up to the study’s start date. Specific keywords related to immunohistochemistry applied in atypical polypoid adenomyoma were used. Search terms included “atypical polypoid adenomyoma” AND “diagnosis” AND “immunohistochemistry” AND “genetics” OR “endometrial adenocarcinoma”. A total of thirty-four relevant scientific articles meeting the inclusion criteria were reviewed: thematic relevance, studies focused on immunohistochemistry, and those providing comparable data for histological differentiation between atypical polypoid adenomyoma and endometrial cancer. For our investigation, we did not use those papers focused on imaging diagnosis, treatment protocols, or clinical features if that information was not related to our areas of interest. Additionally, the ONCOSEGO 2023 guide on endometrial cancer was consulted [10].

### 2.2. Brief Description of Our Patient

A 40-year-old woman presented to the gynecology service with menstrual disturbances and occasional pain, without significant family or personal history. She underwent surgical excision of the endometrium and myometrium. Procedures followed a standardized protocol, including sample fixation in 10% formalin, paraffin embedding, and sectioning into 4 μm thick sections. The initial examination was performed with routine hematoxylin–eosin (H&E) staining, followed by the application of immunohistochemistry (IHC), as described below.

### 2.3. Immunohistochemistry and Meta-Analysis

The initial study based on H&E staining was completed with the application of IHC techniques. For comparison and contrast of results, specific immunohistochemical markers frequently evaluated in the reviewed studies were selected. This study synthesized findings from previous research by compiling works from the literature, with a total of nine articles and 242 cases meeting inclusion criteria, such as immunohistochemical expression in the stroma and glandular epithelium of specific antibodies, like CD10, h-caldesmon, desmin, vimentin, smooth muscle alpha-actin (SMA), b-catenin, Ki67, ER, PR, pancytokeratin, and PTEN; and molecular study of PMS2, MSH2, MSH6, p53, MLH1, and p16.

For the study of our case, IHC was carried out using diluted antibodies as follows: b-catenin (B-CAT-L-CE 1 mL NCL-L-B-CAT), ki67 (KI67-MM1-L-CE 1 mL NCL-L-Ki67-MM1), estrogen receptor (ER-6F11-L-CE 1 mL NCL-L-ER-6F11), progesterone receptor (PGR-312-L-CE 1 mL NCL-L-PGR-312), desmin (DES-DERII-L-CE 1 mL NCL-L-DES-DERII), vimentin (VIM-572-L-CE 1 mL NCL-L-VIM-572), smooth muscle alpha-actin (SMA-L-CE 1 mL NCL-L-SMA), PMS2 (PMS2-L-CE 1 mL NCL-L-PMS2), MSH6 (MSH6-L-CE 1 mL NCL-L- MSH6), MLH1 (MLH1-L-CE 1 mL NCL-L-MLH1), p16 (PA0016 7 mL p16), and p53 (P53-DO7-L-CE 1 mL NCL-L-p53-DO7). Antibody visualization was performed using a detection kit, following the manufacturer’s instructions (Leica Biosystems, Barcelona, Spain)

For the evaluation of immunostaining, a simplification strategy was employed to optimize the comprehension of results obtained from different articles as follows:

- No staining was scored as 0 or negative;
- Staining from 1% to 33% of cells was scored as + (1) (low expression);
- Staining from 34% to 66% of cells was scored as ++ (2) (moderate expression);
- Staining from 67% to 100% was scored as +++ (3) (high expression).

Catenins are cytoplasmic proteins that bind to the highly conserved final portion of the E-cadherin molecule. Beta-catenin is a multiprotein complex of the adherent junction that allows calcium-dependent cell contact, essential for adhesion, signaling, and actin cytoskeleton anchoring. B-catenin acts as an effector of transcription in the Wnt signaling pathway. Positive expression of this molecule, as seen in endometrial cancer, has consequences such as activation of the Wnt signaling pathway, leading to growth, differentiation, and cell proliferation, and is also associated with invasive and metastatic potential of tumor cells, potentially related to higher local invasion and distant spread risk [12].

p16 (INK4a) (cyclin-dependent kinase inhibitor 2A (CDKN2A)) is a tumor-suppressor protein associated with cell cycle progression, specifically regulating the transition from the G1 phase to the S phase. Oncogenic mutations in the CDKN2A gene encoding p16 (resulting in over or underexpression of the protein) are associated with various premalignant and malignant lesions [13,14].

CD10 or neprilysin is a cell-surface metalloendopeptidase that inactivates a variety of biologically active peptides, influencing the regulation of the renin–angiotensin system, the central nervous system, and cell differentiation, and it plays a role in the pathogenesis of certain cancers and other pathological processes [15,16].

Caldesmon is a protein found in smooth muscle cells that regulates muscle contraction and organization of the muscle cytoskeleton. It is mainly expressed in tumors of mesenchymal origin [17,18]. Its role in cancer, including endometrioid cancer, has been studied in relation to its ability to influence cell motility and invasion.

### 2.4. Genetic Analysis

A complementary genetic characterization of our case was performed by next-generation sequencing (NGS) analysis with Action OncoKitDx panel (Health in Code, Valencia, Spain). The Action OncoKitDx is a commercial targeted NGS panel designed for the analysis of genetic alterations in 59 genes relevant in the development of cancer in solid tumors using the NextSeq 550 system (Illumina, San Diego, CA, USA). The genes included in this panel are selected because they have diagnostic, prognostic, and predictive value in the context of targeted therapies (a detailed description of the genes and alterations covered can be found at https://healthincode.com/tumores-solidos-del-adulto-action-oncopaneldx/, accessed on 16 October 2024). Importantly, we use this panel in our daily clinical practice in order to identify potential therapeutic targets for cancer patients. The alterations covered by the panel are divided into mutations (substitutions, insertions, or deletions), alterations in the number of copies (CNVs), and rearrangements. Moreover, the NGS panel integrates a microsatellite instability (MSI) analysis and a pharmacogenetic analysis covering 16 polymorphisms related to main chemotherapy agents. The Action OncoKitDx panel performs hybrid capture-based target enrichment and uses DNA as input for library generation. The NextSeq 550 system performs optical high-throughput sequencing using reversible dye termination sequencing by synthesis technology (paired-end, 2 × 75 pb). The procedure was performed according to manufacturers’ instructions. Automated analysis of sequencing raw data and bioinformatic data analysis was performed by Data Genomics (Health in Code). The bioinformatic analysis includes the alignment of the sequences obtained with the reference sequence (GRCh37/hg19) for the target genes after filtering according to quality criteria, and the identification and annotation of these variants. The Action OncoKitDx panel and the Data Genomics software (IMG-365) are in vitro diagnostic-marked (CE-IVD). The detection limits and technical parameters obtained in the analytical validation of the Action OncoKitDx panel were previously established from samples with the following quality parameters: 200 ng of DNA, DNA integrity number (DIN) > 3, and tumor cell percentage > 50% [19,20].

### 2.5. Data Analysis

Data analysis focused on comparing the expression patterns observed in previous study samples and our case. Qualitative analysis was used to describe the localization and intensity of staining. Results were photographically documented with a microscope, ensuring consistency in lighting and magnification settings. Observed patterns were discussed in the context of findings reported in the literature, using an integrative approach to understand the possible clinical and pathological implications of variations in marker expression.

## 3. Discussion

### Histological Study

**Figure 1:**
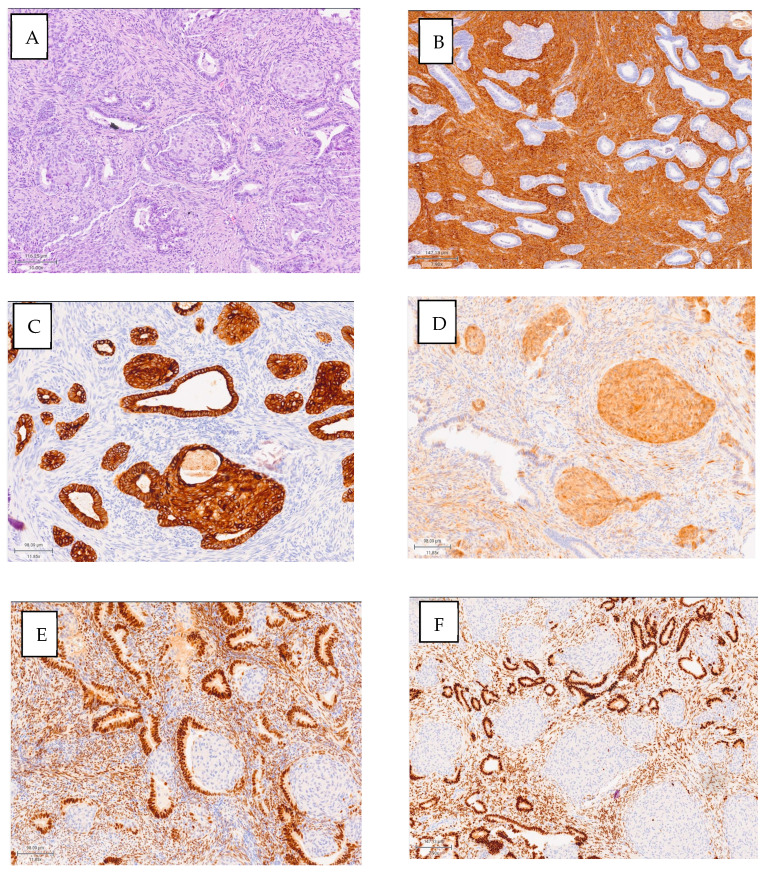
(**A**) Morular, stromal, and glandular area observed (H-E augmentation, 22.50×). (**B**) Actin positive immunostaining of the stroma highlighted (Augmentation 7.90×). (**C**) Keratin AE1–AE3 positivity for glandular lining cells and complete staining of the morula (augmentation, 11.85×). (**D**) p16 positive immunoexpression exclusively in morules (augmentation, 11.85×). (**E**) ER positive in glandular epithelium and intense, weak, or absent stroma in morules (augmentation, 11.85×). (**F**) PR-positive immunostaining in glandular epithelium, sparse in stroma and absent in morules (augmentation, 7.90×).

Several fragments were received, measuring 2.1 × 1.3 cm, and two fragments measuring 0.6 × 0.3 cm, with total inclusion of both, corresponding to a myoma and a polyp, respectively. Histologically, a biphasic tumor composed of endometrioid glands, generally with complex architecture and sometimes cytological atypia, is observed. The glandular component showed a lobulated architecture, benign fibromyomatous stroma, with myxoid change and morular squamous metaplasia. The margin is not evaluable due to the type of resection performed. The sample shows a proliferative endometrial appearance, and our IHC study reveals the following results: nuclear positivity for b-catenin, pancytokeratin, positive ER and PR in glands, positive SMA and desmin in stromal muscle, and positive p16 in squamous morules (Figure 1).

The results shown in the tables are expressed as positive (+), negative (−), or null when the authors did not study the item. In this sense, as stated before, the following should be noted:

- No staining was scored as 0 or negative (−);
- Staining from 1% to 33% of cells was scored as + (1) (low expression);
- Staining from 34% to 66% of cells was scored as ++ (2) (moderate expression);
- Staining from 67% to 100% was scored as +++ (3) (high expression).

It is essential to highlight a significant limitation in the comparative analysis of the obtained data. Considerable variability in reporting methodologies was observed among the analyzed studies. While some authors [1,8,21,22] provided results expressed as a total number of evaluated patients, others limited themselves to report the frequency of positive or negative results [23,24,25]. This discrepancy in data presentation prevents the application of conventional statistical techniques for unified analysis.

Using glandular IHC, depicted in Table 1, the presence of b-catenin was evaluated. Our findings align with those of three other previous studies, which also reported positive b-catenin expression in the analyzed samples. Including our own case, this positive expression pattern has been observed in a total of four studies, reinforcing the consistency and potential importance as a biomarker in glandular tissue. Ki67, as a cell proliferation marker, has been found positive in four studies, although with a low positivity index, as is common in benign tissues. ER and PR show positive results in four studies, along with our own positive findings. Pancytokeratin has shown strong positivity in two studies, similar to our own case. PTEN, a tumor suppressor protein, and its loss may indicate malignant potential, has been analyzed in two studies, resulting in positive outcomes in both. Loss of PTEN expression is commonly observed in endometrial carcinomas, especially in endometrioid types (Table 1).

In the context of stromal IHC (Table 2), vimentin shows positive expression in all cases in two studies, noting that, beyond these mentioned studies, the presence of this biomarker has not been investigated in the rest of the reviewed literature. Desmin has demonstrated strong positivity in four previously published studies, including our own research, confirming its robust expression pattern in a total of five studies, thus highlighting desmin’s reliability as a biomarker in specific glandular tissues. H-caldesmon has been studied in a limited number of investigations, with two out of three articles reporting predominantly negative results, while the third identifies positive expression. Regarding CD10, it was observed negative in two studies and positive in three, with one study showing negative results [1], including a sample size of 99 patients, reinforcing its relevance. Lastly, the presence of the SMA antibody shows notable coherence in the reported findings. Specifically, five previous investigations have consistently documented positive results in the expression of this antibody. Adding our own case, our current study reinforces and extends these findings, corroborating the positive association of the SMA antibody.

In the molecular study analysis reflected in Table 3, the p16 marker showed strong positivity in four studies, including our case. MLH1 and PMS2, studied exclusively in two investigations, resulted in being strongly positive in both cases. Takashi et al. [21] investigated seven APA lesions and found that the CTNNB1 gene was positive for mutations in all cases, suggesting a relationship with β-catenin activity. This finding contrasts with the study by Ota et al. [22], where CTNNB1 mutations were negative in six cases. However, the previous literature shows concordance with the data provided by Takashi.

Table 4 compares the expression of various immunohistochemical markers between atypical polypoid adenomyoma and endometrial adenocarcinoma. CD10 and h-caldesmon were negative in APA, while these markers were positive in EC cases. In contrast, p16 showed positive results in APA and negative in EC. The other markers used (ER, PR, and pancytokeratin) were positive in both cases.

Important findings of the genetic characterization of our case included the presence of a point mutation in exon 3 of CTNNB1 (NM_001904.3) gene [c.94G>T p.(Asp32Tyr)]. No other genetic alterations (including CNVs and rearrangements) were detected by Action OncoKitDx panel, and the sample was microsatellite-stable.

In summary, combining the results from the previous literature studied, along with our case, glandular IHC consistently showed positive expression of b-catenin, Ki67, ER, and PR in four studies. Desmin demonstrated strong positivity in five studies. The molecular marker p16 was strongly positive in four studies, while MLH1 and PMS2 were positive in two. Differences in CTNNB1 gene mutations were observed between APA and EC lesions. Comparison between APA and EC highlighted differences in the expression of CD10, h-caldesmon, and p16.

Online resources from various websites were used for analysis of the variants, such as OncoKB, Human Genome Variation Society, and PharmGKB [26,27,28].

We observed consistent positive expression of β-catenin. This pattern reaffirms its utility as a biomarker in glandular tissue, supporting its role in tumor progression. Similarly, markers such as Ki67, estrogen and progesterone receptors, and pancytokeratin have also shown uniformity in their positive results across multiple studies, reinforcing their reliability and relevance in specific clinical contexts.

Stromal IHC reveals that markers such as vimentin and desmin exhibit strong and consistent positivity, suggesting their value in identifying specific characteristics of stromal tissue. However, the limited research on other biomarkers, such as h-caldesmon and vimentin, in the literature points to an opportunity for additional studies that could explore their role in other pathological conditions.

Regarding the differential diagnosis between APA and EC, both lesions exhibit positive expression for β-catenin, ER, PR, and pancytokeratin, suggesting similarities in underlying cellular pathways. In both endometrioid carcinoma and atypical polypoid adenomyoma, altered expression of β-catenin plays a role in pathogenesis and may serve as a marker for diagnosis and evaluation of malignant potential. However, mutation patterns and expression dynamics may vary, reflecting differences in the underlying biology of these lesions. It is suggested that abnormalities in the β-catenin gene (*CTNNB1*) activate certain signaling pathways (WNT signaling pathways) within the cells. This activation is likely a consequence of genetic mutations and plays a crucial role in how epithelial cells are organized and behave.

As for the difference in the p16 marker, in APA, this positive expression could be related to atypical cellular changes, with partially active cell cycle regulation, in contrast to EC, where negative expression reflects a more advanced state of cell cycle deregulation and malignancy. Research has shown greater stromal p16 reactivity in APA compared to myoinvasive endometrioid carcinoma. Pancytokeratin, in both cases, indicates the epithelial nature of the lesions. H-caldesmon in EC could be involved in tumor progression through its impact on cell mobility, and changes in expression may affect the cells’ ability to invade adjacent tissues and spread to distant areas. It is not expressed in normal endometrial stroma or endometrial stromal neoplasms, making it useful as a specific and sensitive marker in the context of uterine mesenchymal tumors; in the reported series, we can find a high grade of variability which may be due differences in the IHC protocols, such as the choice of antibodies, antigen-retrieval techniques, and staining conditions, and can lead to variable results. Variations in sensitivity and specificity of different antibody clones used may also affect outcomes; as may happen with any other IHC marker, tumors can exhibit heterogeneity, meaning that, within a single lesion, different areas may express markers differently. This biological variability can influence results over different samples or case series. CD10 is a stromal marker and is naturally expressed in normal endometrial stroma, as observed in EC, while in APA, the expression is absent or weakly positive.

Other studies have analyzed the utility of this IHC marker for the diagnosis of APA versus EC. In their cohort of seven APA lesions, only one showed weak focal stromal staining among the glands, unlike the 19 myoinvasive carcinomas with clear positivity. Although the study identifies potential markers for differentiating adenomyoma and adenocarcinoma, additional studies with larger samples are necessary to validate these findings.

We identified the mutation in the CTNNB1 gene [c.94G>A, p.(Asp32Tyr)], which presented an allele frequency of 11%. This finding reinforces the trends observed in similar studies. Additionally, no rearrangements were observed in the analyzed regions, which is in line with expectations based on the pathology under study. The CTNNB1 gene encodes the transcription factor β-catenin, which is involved in the Wnt signaling pathway and plays an important role in cell adhesion and migration processes. Mutations in the CTNNB1 gene have an oncogenic effect and have been described in various solid tumors, including gynecological neoplasms. Therapeutic blocking of the Wnt pathway has been considered for the treatment of various tumors, and evidence suggests that tumors with CTNNB1 gene mutations would require direct pharmacological inhibition of β-catenin, as they would show resistance to treatments targeting components acting at higher levels of the signaling pathway.

## 4. Conclusions

In the differential diagnosis between APA and EEC, the presence of common expression patterns of various biomarkers suggests similarities in their underlying cellular signaling. However, differences in expression dynamics and genetic mutations, especially in genes such as CTNNB1, underscore the distinctions in the biology of these lesions. Additionally, variability in the expression of markers such as p16 and h-caldesmon between APA and EEC reflects their differentiated roles in cell cycle regulation and tumor progression.

Research on atypical polypoid adenomyoma addresses the important diagnostic distinction from endometrioid carcinoma, based on differentiation through immunohistochemical and molecular markers. This differentiation is crucial, as APA, being mostly a benign lesion, allows for conservative clinical management in selected cases, unlike CE, which requires more radical treatments.

This study provides a solid foundation for the use of these biomarkers in clinical diagnosis and suggests areas for future research that could improve our understanding and treatment of these pathological conditions. Additional studies with larger samples are recommended to validate these preliminary findings and potentially discover new diagnostic and therapeutic markers.

## Figures and Tables

**Table 1:** Study of glandular immunohistochemistry in atypical polypoid adenomyoma.

	Glandular Immunohistochemistry					
	No. of Cases	B-Catenin	Ki67	ER/PR	Pancytokeratin	PTEN
**Own case**	1	+	null	+	+	null
**Sun et al. [1]**	99	null	++	+	null	+
**Takashi et al. [21]**	7	+	+	null	null	null
**Nemejcova et al. [8]**	21	++	null	null	null	+++
**Ota et al. [22]**	6	+++	null	null	null	null
**Lu et al. [23]**	36	null	+	+	null	null
**Terada et al. [24]**	5	null	+	+	++	null
**Soslow et al. [25]**	23	null	null	+	+++	null

**Table 2:** Study of stromal immunohistochemistry in atypical polypoid adenomyoma.

	Stromal Immunohistochemistry					
	No. of Cases	CD10-	H-Caldesmon	Desmin	Vimentin	SMA
**Own case**	1	null	null	+	null	+
**Sun et al. [1]**	99	-	-	+++	+++	null
**Takashi et al. [21]**	7	-	null	null	null	+
**Lu et al. [23]**	36	+	-	null	null	+
**Terada et al. [24]**	5	++	null	++	+++	+
**Soslow et al. [25]**	23	null	null	++	null	+++
**Kihara et al. [2]**	12	++	+	++	null	+++

**Table 3:** Molecular study of atypical polypoid adenomyoma.

	Molecular Study						
	No. of Cases	PMS2	MSH6	P53	MLH1	CTNNB1	p16
**Own case**	1	null	null	++	null	+	+
**Nemejcova et al. [8]**	21	++	++	-	+++	null	Null
**Sun et al. [1]**	99	+++	+++	++	null	+	+++
**Takashi et al. [21]**	7	null	null	null	null	null	Null
**Ota et al. [22]**	6	null	null	-	+++	-	Null
**Lu et al. [23]**	36	null	null	-	null	null	Null
**Worrel et al. [4]**	32	null	null	null	null	null	+++
**Kihara et al. [2]**	12	null	null	++	null	null	+++

**Table 4:** Differential diagnosis between atypical polypoid adenomyoma and endometrial adenocarcinoma.

Markers	Atypical Polypoid Adenomyoma	Endometrial Adenocarcinoma
**B-catenin**	+	+
**p16**	+	-
**RE**	+	+
**PR**	+	+
**Pan-cytokeratin**	+	+
**CD10**	-	+
**h-Caldesmon**	-	+

## Data Availability

The data are contained within this article.
